# Supplementary figures and images for: Stress responses upon starvation and exposure to bacteria in the ant Formica exsecta
Source: PeerJ. 2019 Feb 18;7:e6428. doi: 10.7717/peerj.6428 (PMC6383555; doi:10.7717/peerj.6428)

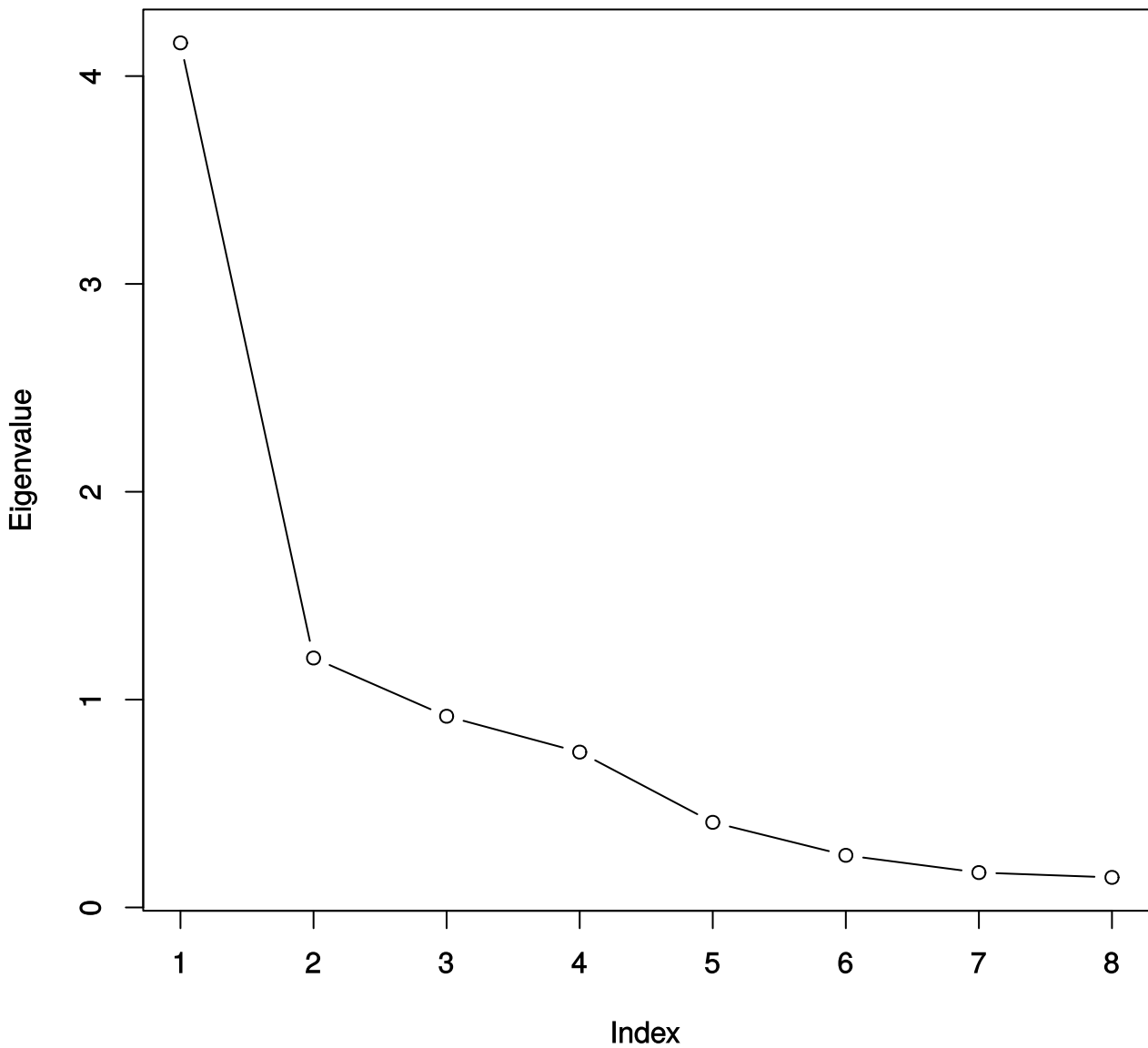

Supplement: Supplemental Information 1 — Scree-plot of the Eigenvalues of each Principal Component from an initial PCA for component selection. [file peerj-07-6428-s001.pdf]

Gene expression (-Ct)

Aryl

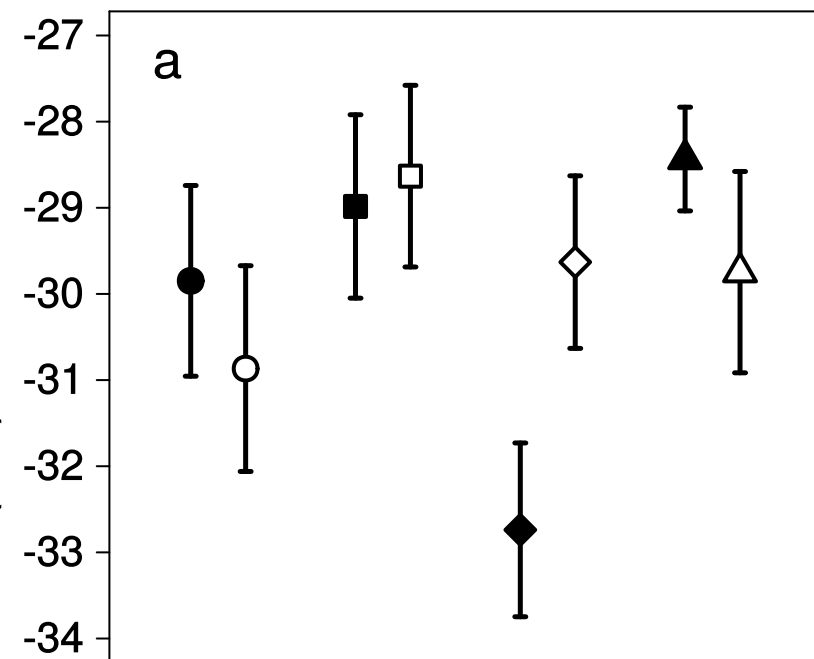

Vg1

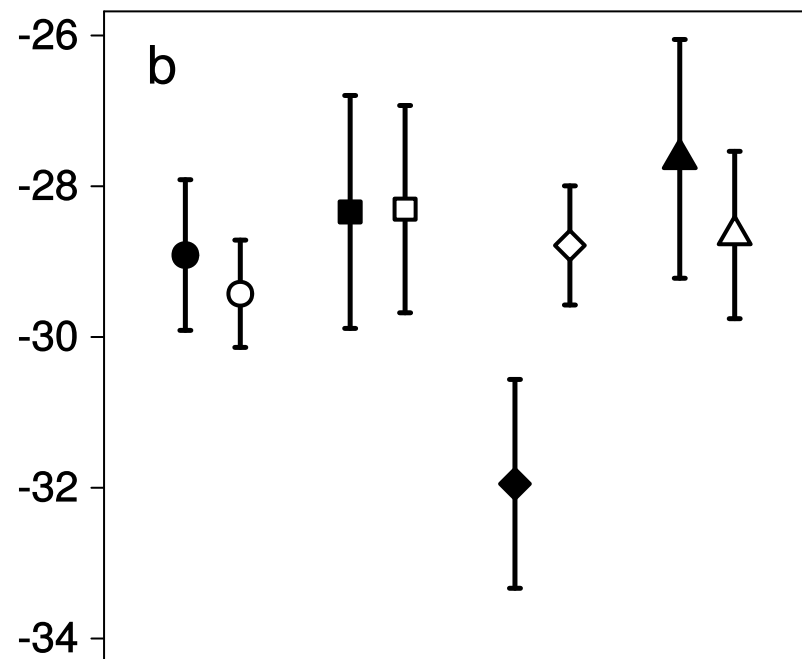

IR3

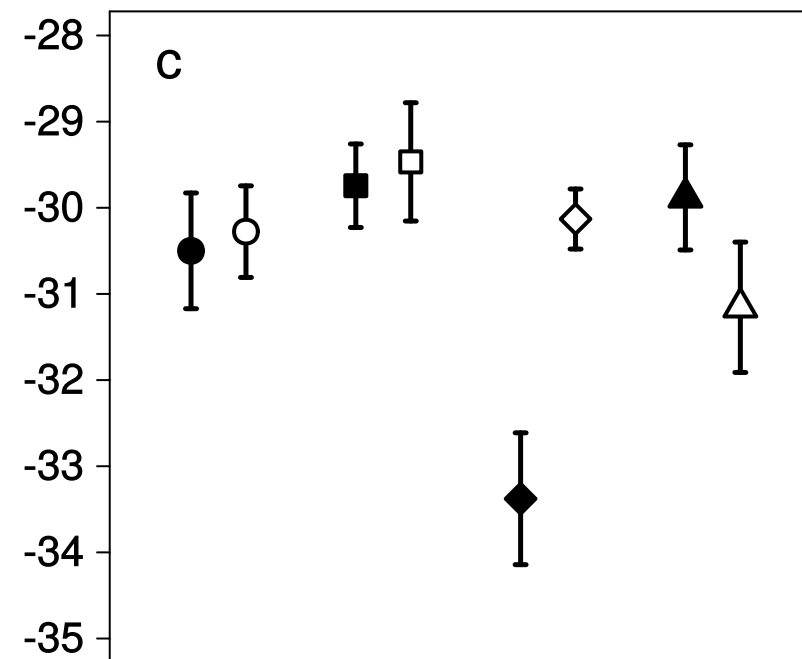

PPO

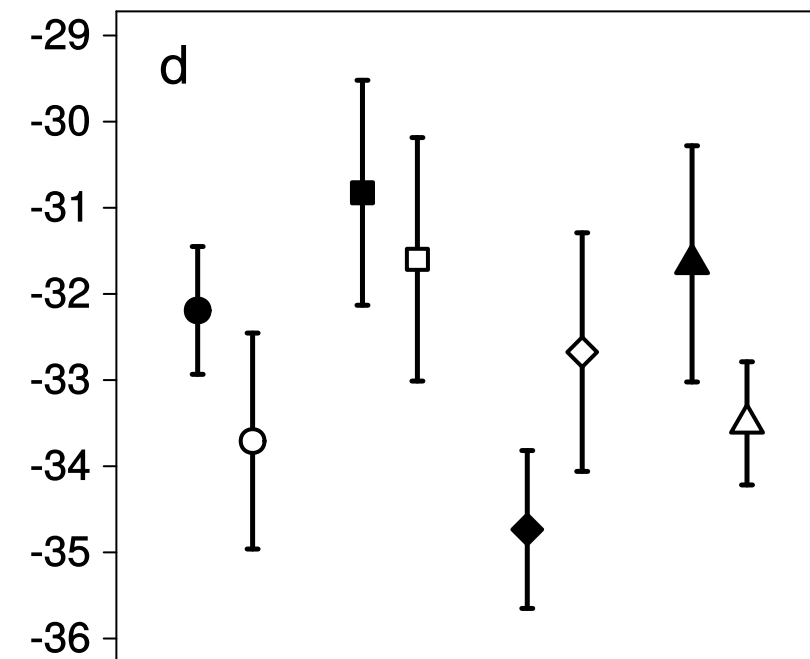

Hyme

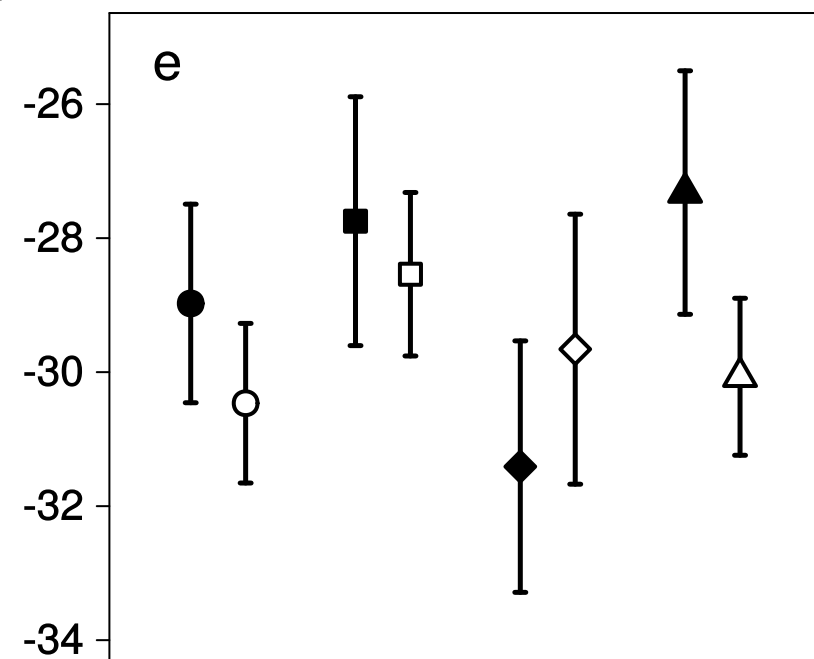

LPS.bp

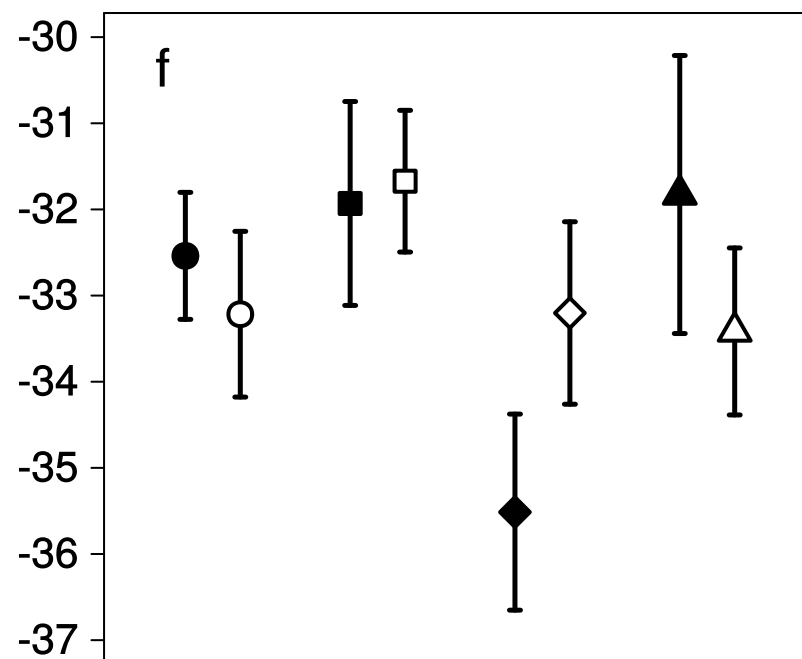

LysC

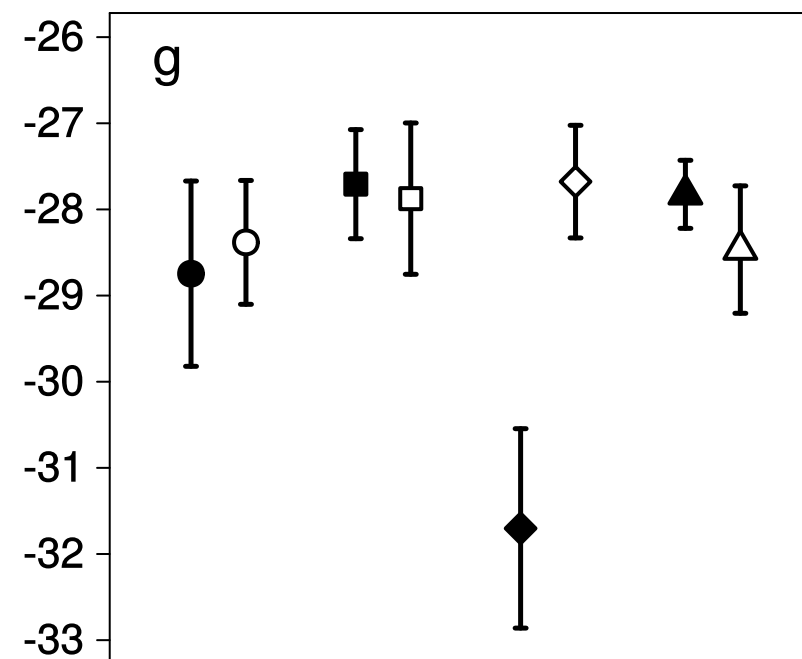

Toll

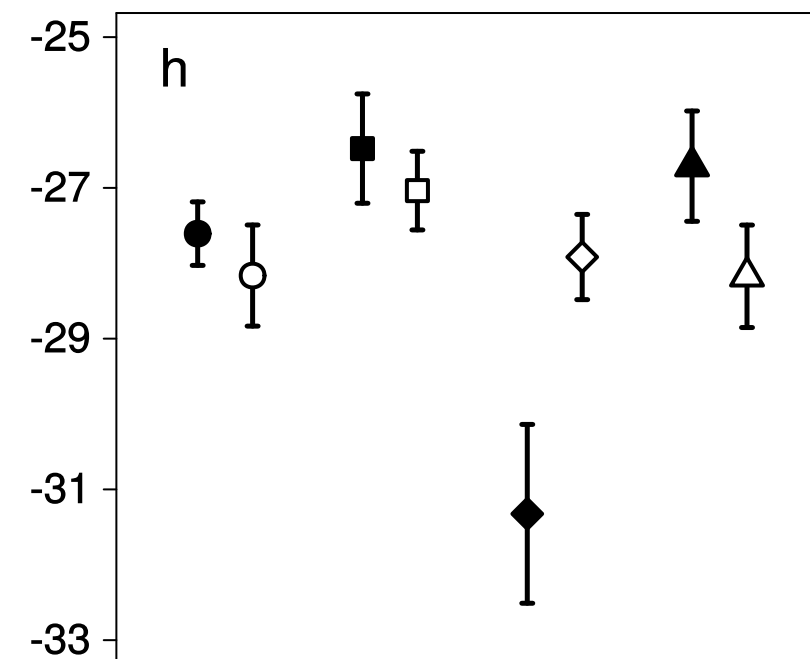

● Continuous ○ Starved

● Co ■ EC ◆ SM ▲ PE

Supplement: Supplemental Information 2 — Average expression (inverted normalized Ct value) of each candidate gene in response to Treatment (Continuous feeding [filled symbols] / Starved [open symbols]) and Diet (Control [circles] / E. coli [squares] / S. marcescens [diamonds] / P. entomophila [triangles]). Error bars indicate 95% confidence intervals. [file peerj-07-6428-s002.pdf]
