# Supplementary material for: Stress responses upon starvation and exposure to bacteria in the ant Formica exsecta
Source: PeerJ. 2019 Feb 18;7:e6428. doi: 10.7717/peerj.6428 (PMC6383555; doi:10.7717/peerj.6428)
Supplement: Supplemental Information 3 — This document contains the Supplementary Tables S1–S5, including captions. [file peerj-07-6428-s003.pdf]

Stress responses upon starvation and exposure to bacteria in the ant *Formica exsecta*

Dimitri Stucki[1,2], Dalial Freitak[1,2], Nick Bos[1,2,3] & Liselotte Sundström[1,2]

1) Organismal and Evolutionary Biology Research Programme, Faculty of Biological and Environmental Sciences, University of Helsinki, PO Box 65 (Viikinkaari 1), FI-00014 Helsinki, Finland

2) Tvärminne Zoological Station, University of Helsinki, FI-10900 Hanko, Finland

3) Section for Ecology & Evolution, Department of Biology, University of Copenhagen, 2100 Copenhagen, Denmark

Supplementary Tables

Table S1: The Primer sequences used for qPCR gene expression analysis.

Gene	Forward	Reverse	Efficiency
Arylphorin	5’-ACT TAG AAC GCC TGT CCA AC-3’	5’-GAG ATT CCA TGT CCT CCA AG-3’	99.9
Hymenoptaecin	5’-TGG CAA AGA CGG TTT CAT CA-3’	5’-AAC CAC CAG TTA CAC CAA CC-3’	102.4
Insulin Receptor 3	5’-GCG AGC GTT TTC TGT AGG TA-3’	5’-CCC AGG AGC TCG ATA ACA AC-3’	105.0
LPS-binding protein	5’-CCA CCG TGT AAC GAT AAT CA-3’	5’-TTG ACG TCA TCG GTC AAT AG-3’	102.9
Lysozyme C	5’-CAC CAT CCT TCT TCT TCG TT-3’	5’-AGG TGG CAT CTG TAA CAA GC-3’	91.6
Pro-Phenoloxidase	5’-TCC AAC TGC TGT AGG CTG TA-3’	5’-ACC TTC ACA ATC TCG GTC AT-3’	96.4
Toll-receptor	5’-GCT CCT TCG TGA CCT AGT CG-3’	5’-GTG AAA GCG CCA TTA TCC AT-3’	102.4
Vitellogenin 1	5’-AGG TTG TCA TCG TCA ACG AA-3’	5’-CGT TGC TTG TAG GGG ATA GG-3’	98.3

Table S2: Eigenvalues and variance from the PCA on gene expression.

	PC1	PC2	PC3	PC4	PC5	PC6	PC7	PC8
Eigenvalue	4.16	1.20	0.92	0.75	0.41	0.25	0.17	0.14
Proportion of Variance	0.52	0.15	0.11	0.09	0.05	0.03	0.02	0.02
Cumulative Variance	0.52	0.67	0.78	0.88	0.93	0.96	0.98	1.00

Table S3: Loadings for PC2 from a preliminary PCA.

Gene	Loading
Arylphorin	-0.36
Vitellogenin 1	-0.67
Insulin Receptor 3	0.02
Pro-Phenoloxidase	0.48
Hymenoptaecin	0.29
LPS-binding protein	0.53
Lysozyme C	-0.14
Toll-receptor	-0.02

Table S4: Results for PC2 from a preliminary linear mixed effects model on PC2-scores.

	β±SE	t	p-value
Starved	-0.34±0.47	-0.71	0.48
E. coli	0.17±0.47	0.37	0.72
S. marcescens	0.05±0.47	0.11	0.92
P. entomophila	-0.20±0.47	-0.42	0.67
Starved**E. coli*	0.11±0.67	0.16	0.87
Starved**S. marcescens*	-0.17±0.67	-0.26	0.80
Starved**P. entomophila*	-0.16±0.67	-0.23	0.82

Table S5: Results from separate pairwise comparisons of gene expression for each gene. Comparisons were performed between Treatments (Continuously exposed (C), Starved (S)) within each Exposure regime, as well as between each bacterial Exposure regime (Control exposure (Co), *S. marcescens* (SM), *E. coli* (EC), *P. entomophila* (PE)) within each Treatment. All p-values were adjusted for multiple comparisons using false discovery rates.

	Aryl			Vg1			IR3			PPO		
	β±SE	z	adj.p	β±SE	z	adj.p	β±SE	z	adj.p	β±SE	z	adj.p
C/Co vs S/Co	1.02±0.64	1.60	0.5551	0.51±0.71	0.72	0.6371	-0.22±0.38	-0.59	0.6371	1.52±0.70	2.16	0.4925
C/EC vs S/EC	-0.35±0.64	-0.55	0.9584	-0.04±0.71	-0.05	0.9584	-0.28±0.38	-0.73	0.9584	0.77±0.70	1.10	0.9584
C/SM vs S/SM	-3.11±0.64	-4.88	0.0000	-3.16±0.71	-4.43	0.0001	-3.25±0.38	-8.54	0.0000	-2.06±0.70	-2.93	0.0123
C/PE vs S/PE	1.31±0.64	2.06	0.1203	1.01±0.71	1.41	0.3601	1.28±0.38	3.36	0.0120	1.85±0.70	2.64	0.0509
C/Co vs C/EC	-0.86±0.64	-1.36	0.3454	-0.57±0.71	-0.80	0.6161	-0.76±0.38	-1.99	0.2384	-1.37±0.70	-1.95	0.2384
S/Co vs S/EC	-2.23±0.64	-3.50	0.0117	-1.12±0.71	-1.57	0.3542	-0.81±0.38	-2.13	0.0943	-2.11±0.70	-3.01	0.0425
C/Co vs C/PE	-1.41±0.64	-2.22	0.2950	-1.28±0.71	-1.79	0.3167	-0.62±0.38	-1.63	0.3167	-0.54±0.70	-0.77	0.4976
S/Co vs S/PE	-1.12±0.64	-1.76	0.5151	-0.78±0.71	-1.09	0.9824	0.88±0.38	2.31	0.3343	-0.21±0.70	-0.29	0.9824
C/Co vs C/SM	2.89±0.64	4.53	0.0000	3.04±0.71	4.25	0.0001	2.88±0.38	7.57	0.0000	2.54±0.70	3.62	0.0018
S/Co vs S/SM	-1.24±0.64	-1.94	0.8221	-0.64±0.71	-0.90	0.8247	-0.15±0.38	-0.39	0.8247	-1.03±0.70	-1.47	0.8221
C/EC vs C/SM	3.75±0.64	5.89	0.0000	3.61±0.71	5.05	0.0000	3.63±0.38	9.56	0.0000	3.91±0.70	5.57	0.0000
S/EC vs S/SM	1.00±0.64	1.57	0.3135	0.48±0.71	0.67	0.7042	0.66±0.38	1.74	0.3135	1.08±0.70	1.53	0.3200
C/EC vs C/PE	-0.55±0.64	-0.86	0.9350	-0.70±0.71	-0.99	0.9350	0.14±0.38	0.36	0.9350	0.83±0.70	1.18	0.9350
S/EC vs S/PE	1.11±0.64	1.75	0.2060	0.34±0.71	0.48	0.7213	1.69±0.38	4.44	0.0002	1.90±0.70	2.71	0.0573
C/SM vs C/PE	-4.30±0.64	-6.75	0.0000	-4.31±0.71	-6.03	0.0000	-3.50±0.38	-9.20	0.0000	-3.08±0.70	-4.39	0.0001
S/SM vs S/PE	0.12±0.64	0.18	0.9031	-0.14±0.71	-0.19	0.9031	1.02±0.38	2.70	0.1286	0.83±0.70	1.18	0.7868
	Hyme			LPS-bp			LysC			Toll		
	β±SE	z	adj.p	β±SE	z	adj.p	β±SE	z	adj.p	β±SE	z	adj.p
C/Co vs S/Co	1.49±0.98	1.51	0.5551	0.68±0.67	1.01	0.6371	-0.36±0.50	-0.73	0.6371	0.56±0.44	1.26	0.5551
C/EC vs S/EC	0.79±0.98	0.81	0.9584	-0.26±0.67	-0.39	0.9584	0.17±0.50	0.34	0.9584	0.56±0.44	1.26	0.9584
C/SM vs S/SM	-1.75±0.98	-1.78	0.1991	-2.31±0.67	-3.46	0.0029	-4.03±0.50	-8.07	0.0000	-3.41±0.44	-7.71	0.0000
C/PE vs S/PE	2.75±0.98	2.80	0.0551	1.59±0.67	2.38	0.0708	0.64±0.50	1.29	0.3601	1.46±0.44	3.31	0.0120
C/Co vs C/EC	-1.23±0.98	-1.25	0.4516	-0.61±0.67	-0.91	0.6032	-1.04±0.50	-2.08	0.2384	-1.13±0.44	-2.55	0.1718
S/Co vs S/EC	-1.92±0.98	-1.96	0.2143	-1.54±0.67	-2.31	0.0885	-0.51±0.50	-1.02	0.4492	-1.13±0.44	-2.55	0.0573
C/Co vs C/PE	-1.66±0.98	-1.68	0.3167	-0.71±0.67	-1.07	0.4976	-0.92±0.50	-1.85	0.3167	-0.90±0.44	-2.03	0.2950
S/Co vs S/PE	-0.39±0.98	-0.40	0.9824	0.20±0.67	0.30	0.9824	0.08±0.50	0.17	0.9824	0.01±0.44	0.02	0.9824
C/Co vs C/SM	2.44±0.98	2.48	0.0530	2.97±0.67	4.45	0.0001	2.96±0.50	5.93	0.0000	3.72±0.44	8.42	0.0000
S/Co vs S/SM	-0.81±0.98	-0.82	0.8247	-0.01±0.67	-0.02	0.9831	-0.71±0.50	-1.41	0.8247	-0.25±0.44	-0.56	0.8247
C/EC vs C/SM	3.66±0.98	3.73	0.0016	3.58±0.67	5.36	0.0000	4.00±0.50	8.01	0.0000	4.85±0.44	10.97	0.0000
S/EC vs S/SM	1.12±0.98	1.14	0.4949	1.53±0.67	2.29	0.2950	-0.20±0.50	-0.40	0.8412	0.88±0.44	1.99	0.2950
C/EC vs C/PE	-0.43±0.98	-0.43	0.9350	-0.10±0.67	-0.16	0.9350	0.12±0.50	0.24	0.9350	0.23±0.44	0.53	0.9350
S/EC vs S/PE	1.53±0.98	1.56	0.3080	1.74±0.67	2.61	0.0582	0.59±0.50	1.19	0.4315	1.14±0.44	2.57	0.0573
C/SM vs C/PE	-4.09±0.98	-4.16	0.0005	-3.69±0.67	-5.51	0.0000	-3.88±0.50	-7.77	0.0000	-4.61±0.44	-10.44	0.0000
S/SM vs S/PE	0.41±0.98	0.42	0.9031	0.21±0.67	0.32	0.9031	0.79±0.50	1.58	0.7868	0.26±0.44	0.58	0.9031
